# Effect of Various Cooking Methods on Technological and Sensory Quality of Atlantic Salmon (*Salmo salar*)

**DOI:** 10.3390/foods8080323

**Published:** 2019-08-07

**Authors:** Artur Głuchowski, Ewa Czarniecka-Skubina, Grażyna Wasiak-Zys, Dorota Nowak

**Affiliations:** 1 Department of Food Gastronomy and Food Hygiene, Faculty of Human Nutrition and Consumer Sciences, Warsaw University of Life Sciences (WULS), 02-787 Warsaw, Poland; 2 Department of Functional Food, Ecological Food and Commodities, Faculty of Human Nutrition and Consumer Sciences, Warsaw University of Life Sciences (WULS), 02-787 Warsaw, Poland; 3 Department of Food Engineering and Process Management, Faculty of Food Sciences, Warsaw University of Life Sciences (WULS), 02-787 Warsaw, Poland

**Keywords:** salmon, sous-vide cooking, sensory quality, consumer testing, texture, color

## Abstract

The aim of the study was to evaluate the effect of heat treatment salmon quality using the sous-vide method (57 °C, 20 min and 63 °C, 80 min) in comparison with traditional methods (steam cooking, roasting). The yield of process and sensory quality (sensory profile, consumer liking) and the color and texture of salmon was measured. Salmon processed with the sous-vide method was characterized by a statistically significantly (*p* ≤ 0.05) higher yield and water content than the samples prepared by steaming and roasting. Statistically higher (*p* ≤ 0.05) consumer preference for salmon prepared using higher parameters of the sous-vide process (63 °C, 80 min) compared with low parameters (57 °C, 20 min) was stated. Parameters of the sous-vide processing (57 °C, 20 min) have a negative effect on salmon quality. It was observed that as the temperature and time of the process increased, the intensity of the flavor and odor attributes of cooked fish also increased, while the intensity of raw fish, juiciness, tenderness, and softness decreased. The color of salmon also changed. Based on the results, we recommend the following parameters of process in preparing salmon using the sous-vide method: 63 °C for 80 min.

## 1. Introduction

The sous-vide method is defined as “cooking under controlled conditions of temperature and time inside head stable vacuumed pouches.” Sous-vide products (raw materials or its combination with semi-finished products) are heated under mild temperatures, mostly at temperature range of 65–95 °C for a longer period of time [1]. Scientific interest initially focused on its usefulness in extending a product’s shelf life using pasteurization dose, thereby ensuring food safety. The sous-vide method was also used as a method of cooking in the previous decade [2,3].

The sous-vide method gives fish a fresh and natural-like appearance, but too high temperature leads to a decrease in its sensory quality. Although the recommended heat treatment parameters in sous-vide fish processing oscillates in a range of 60–80 °C for 20–40 min. [1], in practice, a lower temperature (40–60 °C) is used. Traditional heat treatment methods are performed to reach core temperature of about 55–65 °C. To achieve the most preferred texture and flavor, some authors recommend a maximum core temperature of about 40 °C [2]. This method is called “novel sous-vide” (low temperature, short time), and it has its enthusiasts [4]. Low process parameters have a positive effect on flavor, increased juiciness, and reduced thermal shrinkage of the product, but it results in different degree of doneness, raw-like appearance of the core, and can also pose a microbiological hazard [5]. According to Nieva-Echevarria et al. [6], the volatile compounds profile of fish (sea bass) processed with the sous-vide method of cooking was similar to that processed with the steamed method.

In the case of fish, texture is an important trait of quality, thereby process temperature seems to be the key parameter. Temperature also determines microbiological stability of fish during storage under refrigeration conditions. For example, although, cooking at 70 °C results in a better for the texture of collagen-rich fish, it may not be sufficient to ensure safety when stored for a longer period of time [7,8]. The influence of different factors such as flavor additives (spices, lemon juice, sauces), application of complex methods, and storage conditions on the sensory quality of fish [1,2,4,5,9,10,11,12,13,14,15,16,17,18,19,20,21,22] and seafood [8,23,24,25,26,27] prepared with the sous-vide method has been investigated by several authors. Only a few studies focused on salmon [1,2,11,17,18,20,22,26].

Previous research mostly focused on the use of this method in the preparation and chilled storage of dishes as well as in their microbiological quality. Due to the growing popularity of sous-vide as a method of cooking in restaurants, some studies have aimed to determine the effect of the sous-vide process on the sensory quality of dishes (without storage), including research on regional species of fish (whiting, bonito, carp, tuna) or shellfish [2,8,14,15,16,24,25]. No studies were devoted to assessing both the sensory profile and hedonic response to fish processed with sous-vide method, especially when compared with those prepared traditionally. So far, the sous-vide method for storing products and extending their shelf life has been used. Today, the method is also used as a sensory-motivated cooking method. In modern food service, where zero-waste cooking and food waste are becoming more and more important, it can be an alternative to traditional cooking methods.

The aim of the study was to assess sensory quality and instrumental color and texture of sous-vide salmon processed with different parameters (novel sous-vide, pasteurization dose) in comparison with conventional dishes (steaming and roasting).

## 2. Materials and Methods

### 2.1. Material

The Atlantic salmon *(Salmo salar)* was delivered directly by a distributor from Norway (Fiord S.C., Józefów, Poland), with cold chain being maintained, and then stored according to the producer’s recommended temperature of 3 ± 1 °C. A whole side fillet prepared in accordance with GMP guidelines was portioned, calibrated to a height of 25 ± 2 mm, and weighed. The average portion of the salmon fillet was 276 ± 21 g.

### 2.2. Heat Treatment Methods

As a reference point for the sous-vide method, roasting en papillote and steaming were selected, due to significant use of these heat treatment methods in the catering industry for salmon preparation. In the sous-vide method, two parameters of thermal processing were selected in preliminary research, based on Baldwin [3] calculation and suggested by the sous-vide equipment producer. Initial process parameters from manufacturer’s guideline (52 °C 20 min.) did not meet with sensory panel acceptance due to the lack of cooked fish traits, and it was increased to 57 °C to gain approval. En papillote made from aluminum foil (250 × 290 mm) was used to limit the formation of Maillard compounds that could affect the higher degree of salmon overall liking. Due to the delicate structure of salmon, a 60% pressure reduction was applied. The conditions of heat treatment methods are presented in Table 1.

The process yield and water content of salmon fillets processed with various heat treatment methods were determined.

### 2.3. Technological Parameters of Quality

Yield was calculated with a weight method as a difference in weight before (raw) and after cooking, based on 12 repetitions. Determination of water content was carried out in accordance with [29].

### 2.4. Sensory Evaluation and Consumer Testing

Analytical evaluation of sensory quality was carried out using the quantitative descriptive method, while consumer tests were conducted using questionnaire and hedonic scale.

#### 2.4.1. Sample Preparation and Presentation

All samples of salmon (20 g) up to 5 min after heat treatment were portioned to plastic containers, then covered with a lid, and encoded. Thereafter, they were placed in thermally insulated Styrofoam boxes to maintain the temperature (50 ± 1 °C) and delivered for evaluation. Temperature was measuring before sensory analysis.

#### 2.4.2. Sensory Analytical Method

The sensory quality of cooked salmon was evaluated by 10 persons assessing panel using a quantitative descriptive analysis. Participating members were qualified experts according to [30]. Conditions of the assessment were determined in accordance with Civille and Carr [31]. Selection of quality descriptors for quantitative descriptive analysis was carried out in accordance with the ISO procedure [32]. To determine intensity of each sensory attribute, an unstructured graphical scale of 100 mm (0–10 conventional units) with specific word anchors was used.

The following five attributes were evaluated: (1) odor: cooked fish, raw fish/seaside, fish oil, roasty, muddy/musty, “other” odor (with word anchors none–strong); (2) color intensity with marginal determinations: light–dark; (3) fish texture: fragmentation ability (of myoseptum) with definitions clumps fragmented to myoseptum, hardness (soft–hard), juiciness (none–very juicy), as well as intensity of cellular juice and fat leakage (not visible–very visible); (4) flavor: cooked fish, raw fish, roasted fish, salty taste, fatty, “other” flavor with word anchors none–strong; (5) overall quality. Overall quality as the general degree of individual quality characteristics harmonization (bad–very good) was also assessed. Panelists regularly received encoded samples, a scorecard along with definitions of evaluated attributes, and water to clean the taste buds between sessions. Evaluation was repeated three times.

#### 2.4.3. Testing Conditions

Consumer tests consisted of a validated seven-part questionnaire and hedonic rankings. The respondents were asked about their frequency of salmon consumption, whether they liked it, and the most frequently eaten forms (raw, smoked, cooked). They were also asked to indicate whether they prepared salmon themselves and what methods of heat treatment they usually use. Then, they received fish samples and assessed them. To quantify consumer degree of salmon liking (odor, appearance, flavor, texture, overall), the nine-point classical hedonic scale (from 9—like extremely to 1—dislike extremely) was used [33,34]. The assessment of overall liking with hedonic scale was verified by the ranking method, where salmon samples were ranked by assessors from the most (1) to the least (4) liked [31,34]. Evaluation of salmon dishes was carried out in a group of 120 consumers from specific university population (students, academics), dominated by women (75.8%) in age range of 19–25 (70.8%). A smaller percentage (19.2%) of consumers were between the ages of 26–55 years. The participants in the research were a convenient sample of consumers.

### 2.5. Instrumental Color and Texture Measurements

Instrumental color measurements were made using a spectrophotometer (CM-2300d, Konica-Minolta GmbH, Langenhagen, Germany). The equipment was set up for standard illuminate D_65_ (10° observer angle) and calibrated using a standard white reflector plate (Minolta Technical Note 1994). The measurement was performed in three replications in raw salmon and after heat treatment. Results were expressed as L* (lightness), a* (redness/greenness), and b* (yellowness/blueness) in the CIE Lab system. Differences (Δ) between given coordinates were calculated by subtracting the colorimetric values of heat-treated sample from the raw material values. The values of color parameters were calculated using the following formulas:

Saturation delta chroma

(ΔC) = √((Δa*)^2^ + (Δb*)^2^)

The chroma with (Intensity of color)

C* = √[(a*)^2^ + (b*)^2^]

The value of total color difference

ΔE*_ab_ = √[(ΔL*)^2^ + (Δa*)^2^ + (Δb*)^2^]

The texture parameters were measured using Texture Analyser TA-XT2 (Stable Micro Systems Ltd., Godalming, UK) after thermal treatment and calibration to room temperature at 23 °C. Due to the complexity of the salmon muscle structure, two shearing tests were performed. The first, using Warner-Bratzler (HDP/BS) shear force attachment, was performed on a sample of salmon cube of side 2.5 ± 0.05 cm. The test parameters were as follows: pre-speed test 1 mm/s, speed test 1.0 mm/s, distance of deformation is 20 mm. The second test was performed using the Craft blade (A/CKB), on single myoseptum with the following dimensions: width 2.5 ± 0.05 cm and thickness of 0.5 ± 0.02 cm. The test parameters were as follows: pre-speed test 0.5 mm/s, speed test 0.5 mm/s, distance of cutting 5 mm. In both tests, the force–distance measurements were performed in 10 repetitions. Based on obtained cutting curves, the maximum cutting force (as a maximum peak force) as a measure of hardness and the work of shear as the total positive area under the curve were determined. Data were analyzed using Texture Expert software version 1.19 for Windows (Stable Micro Systems Ltd, Godalming, UK).

### 2.6. Statistical Analysis

The Shapiro-Wilk test was performed to verify the normality of distribution. One-way analysis of variance (ANOVA) for normal data distribution (sensory profile, yield, and water content). The Kruskal-Wallis test was performed for abnormal data distribution (consumer assessment). A coefficient of correlation according to Pearson calculation and Spearman’s rho was performed with the statistical package STATISTICA software version 13.1 PL (StatSoft, Krakow, Poland). Interpretation of the sensory results obtained was carried out using the Principal Component Analysis (PCA) in accordance with Borgognone et al. [35].

## 3. Results

### 3.1. Water Content and Yield of Process

The yield of heat treatment methods was varied depending on the parameters used. Salmon processed with the sous-vide method was characterized by a higher yield (90.9–93.7%) than the roasted en papillote and steamed (83.8 and 88.4%) methods. This was reflected through a higher water content in these samples (Table 2). The highest yield (93.7%) of the process was obtained in sous-vide salmon SV_57_ (57 °C, 20 min), while the smallest yield in roasted one (83.8%). It was found that as the process temperature increased, the yield and water content in the salmon fillets decreased (SV_57_ < SV_63_ < SP_100_ < RP_180_). The sous-vide process was also characterized by higher repeatability of process results, when compared with traditional methods.

### 3.2. Effect of Various Heat Treatment Methods on Sensory Profiles

Along with the increase in the process parameters, SV_57_ < SV_63_ < SP_100_ < RP_180_), the intensity of cooked and roasted fish odors and flavor, hardness and fragmentation ability increased, while the intensity of salmon color, juiciness, and an intensity of cellular juice leakage decreased. Most odor and flavor attributes of salmon SV_63_ (63 °C, 80 min) that are typical of cooked fish had similar or even higher intensity than the steamed and roasted samples (SP_100_ and RP_180_). It only differed significantly (*p* ≤ 0.05) in lower intensity of cooked fish odor (5.8 c.u.—conventional unit) and higher juiciness (6.7 c.u.). Salmon processed via the sous-vide method SV_57_ (57 °C, 20 min) had the most varied sensory profile from the traditionally made samples (SP_100_ and RP_180_).

No significant differences (*p* > 0.05) between the analyzed samples in intensity of raw fish/seaside odor and flavor, fish fat odor, muddy/must odor, “other” (undefined precisely by panelists) odor and flavor, salty taste, and fatty flavor has been stated. The highest overall quality (7.8) was of steamed salmon (SP_100_), which differed significantly (*p* ≤ 0.05) from the sous-vide sample prepared at 57 °C (6.9 c.u.) but did not differ (*p* > 0.05) from other samples (sous-vide (SV_63_) and roasting (RP_180_)). The lack of differences in the intensity of the salmon color between roasted and SV_63_ samples according to panelists (Table 3) was reflected in instrumental measurements.

In both cases, a similar thermal pasteurization dose was used, as well as a factor limiting the contact of fillet with air and heating medium (aluminum foil, vacuum packaging bags). The decrease in juiciness of salmon samples correlates positively with yield (*r* = 0.48, *p* ≤ 0.05), while hardness correlates (*r* = 0.84, *p* ≤ 0.05) with the water content in the samples.

**Figure 1:**
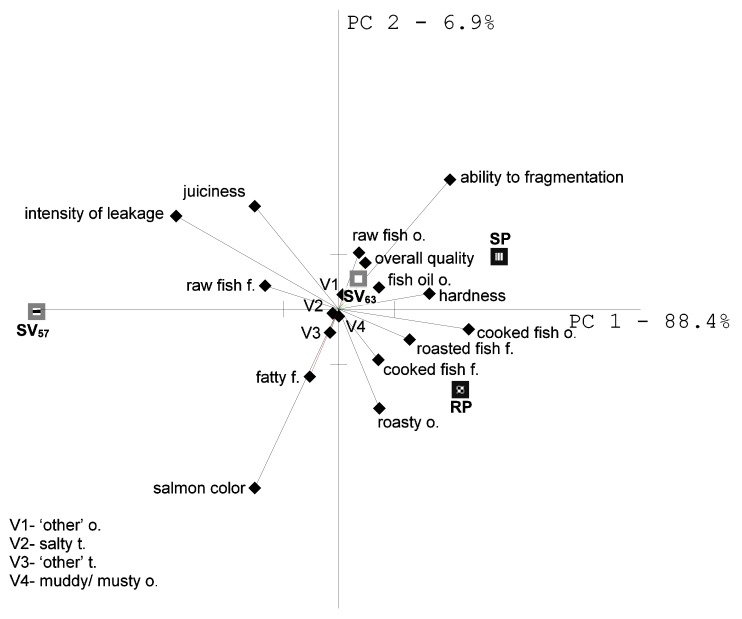
Sensory profile of salmon processed with various heat treatment methods (PCA biplot). SV_57_ (57 °C, 20min), SV_63_ (63 °C, 80min)—sous-vide methods, SP_100_—steaming, RP_180_—roasting en papillote, (o.—odor, f—flavor, t—taste).

The first two principal components of PCA explained 95.3% of the total variance between samples (Figure 1). In general, samples of cooked salmon were mainly differentiated in texture attributes and color as evidenced by the length of their vectors. Fragmentation ability was maximally negatively correlated with salmon color and fatty and “other flavor,” which is proven by the location of those attributes on the opposite side of the plot origin. The intensity of salmon color was positively correlated with intensity of fatty and “other” flavor. The results of PCA analysis indicated that the intensity of roasted fish flavor was not correlated with salmon color, which reflects the angle (α = 90°) between positions of those vectors.

Salmon processed with sous-vide method SV_63_ and the steamed method (SP_100_) were characterized by similar sensory profile, as evidenced by the position of those samples at the same quadrant. Both samples were characterized by the lower notes of raw fish and fish fat odors, as well as higher hardness and fragmentation ability compared with SV_57_ sample (Table 3, Figure 1).

Sensory quality of salmon roasted en papilotte (RP_180_) is associated with high intensity of cooked and roasted flavors and odors. Sous-vide salmon processed with the lowest parameters (SV_57_) was mostly represented by the different texture of the fish than the other ones, i.e., higher intensity of cellular juice and fat leakage and high juiciness, as well as high juiciness and low hardness. It was also characterized by darker tone of salmon color intensity and higher notes of raw fish flavor when compared with other samples. Although higher juiciness and intense salmon color of the fillet are desirable sensory attributes, it indicates lack of cooked fish traits and explains the lower overall quality of the sample evaluated by the sensory panel.

### 3.3. Effects of Various Heat Treatment Methods on Sensory Quality—Consumer Assessment

Consumers surveyed (*n* = 120) declared that they like salmon (92.6%), and most of them (93.4%) consumed it after processing and cooking, including 39.7% of them who preferred the smoked form (not shown in the tables. A significant share of the respondents (71.9%) prepared the salmon fillet themselves. As the most commonly used heat treatment for salmon processing, oven roasting (49.2%) and pan frying (43,3%) were indicated, while grilling (27.5%) and steaming (18,3%) were less popular. A smaller percentage of respondent (7.4%) process the salmon using other methods (in a steamer, in a microwave oven, cooking in a pot in water)—7.4%. Only two people used the sous-vide method for salmon preparation (1.7%). It should be mentioned that the method is used mainly in the food service industry and rarely in domestic kitchens. Respondents declared relatively frequent salmon consumption. Over one in five people (20.7%) did not eat salmon at all or ate it less than once a month. Almost half of the respondents ate salmon once a month (49.6%), 27.3% once a week, and less than 2% every day.

A significant positive correlation between the frequency of salmon consumption and the most commonly used heat treatment methods was stated (*p* ≤ 0.05). Significantly more people who consume a salmon fillet once a week use the oven roasting (*r* = 0.19) or grilling (*r* = 0.26) method to prepare a salmon. A similar correlation between people who declare the cooked salmon consumption as the most frequently consumed form and use of the roasting (*r* = 0.21) or grilling (*r* = 0.29) during salmon preparation was observed.

All prepared samples in the opinion of consumers (*n* = 120) were slightly liked (>6 c.u.), as shown in Table 4. The degree of liking of sous-vide salmon differed depending on the given parameters. The SV_57_ sample was characterized by significant lower hedonic scores for odor, texture, flavor, and overall liking than in other samples. Although the hedonic scores for appearance, flavor, and overall liking of the SV_63_ sample were significantly higher than SV_57_ and steamed samples, they were not different from the roasted en papillote (*p* ≤ 0.05). Among traditional heat treatment methods, the steamed salmon was rated lower by consumers. Significant (*p* ≤ 0.05) correlations between overall liking and flavor-liking (rho = 0.9; *p* ≤ 0.05) or texture-liking (rho = 0.82; *p* ≤ 0.05) were revealed. Degree of liking of odor and appearance had lower effect. There were no statistically significant differences in salmon assessment between gender and age of assessors (*p* > 0.05).

### 3.4. Effect of Various Cooking Methods on Color and Texture of Instrumental Measurement

The results of instrumental color measurement (Table 5) showed tendency that, along with increasing process temperature, the brightness (L*) of the fillets increased, while redness (a*) and yellowness (b*) decreased, resulting in the lower color chroma (C*).

However, the color of the SV_57_ sample varied from others. The sample (SV_57_) was the darkest (ΔL = 23.90), as well as more red (Δa = 1.71) and yellow (Δb = 6.14) when compared to raw salmon. The total color difference (ΔE) between thermal treated and raw salmon of SV_57_ was only 24.73, while ΔE of other samples oscillated within 33.47–34.4. The color of sample SV_63_ was the most similar steamed salmon. The total color difference between these samples amounted to ΔE = 1.66, while the difference between traditional methods (roasting, steaming) was slightly higher ΔE = 2.14 (data not shown in the table). This was reflected in the sensory profile.

During panel discussion, the experts indicated a notable color difference between sous-vide salmon (SV_57_) and other samples. Additionally, a lower amount of coagulated protein on the SV_57_ sample surface was underlined, while the highest was on the steamed salmon surface. It was reflected in shear force, which is stated in the instrumental analysis.

The results of instrumental texture measurements of salmon subjected to different heat treatments revealed that the lowest shear force (3.9 N) was applied to sous-vide salmon prepared at 63 °C for 80 min (SV_63_) (Table 6). The duration of thermal treatment in conditions of high humidity resulted in the hydrolysis of collagen and the disintegration of myofilaments, resulting in a small interaction with the knife during instrumental measurement.

Sous-vide salmon processed at 57 °C (SV_57_) and roasted salmon (RP_180_) require a higher shear force of 5.9 N and 5.8 N (Newton), respectively. Shorter processing time and lower temperature limited collagen hydrolysis for SV_57_ material, and only a part of myofibrin proteins could be denatured (coagulation temperature of 26–52 °C) [36]. On the other hand, during roasting in en papillote, the coagulation of proteins could be limited by the lower humidity of the environment (in this sample, the highest culinary loss was noted—16.2%). In addition, the air layer between fish and aluminum foil simultaneously reduced the heat penetration and the temperature of the fish surface, thereby reducing coagulation. Under these conditions, sarcoplasmic proteins may also coagulate, as their coagulation temperature ranges between 47–78 °C [36]. The highest shear force (6.9 N) was used for a sample of steamed salmon. In these process conditions, the surface reaches the vapor temperature (100 °C) immediately, which caused the coagulation of the most thermal-resistant proteins, i.e., actinin, which leads to the toughening of samples. In the case of texture analysis of individual myofilaments, the degree of proteins coagulation should also affect the maximum shear force. The highest shear force was observed in the case of SV_63_ sample, probably due to the longest processing time. However, this value did not differ significantly from the shear force of roasted salmon.

## 4. Discussion

### 4.1. Technological Quality

The low process temperatures (57 and 63 °C) of sous-vide method, despite its longer processing time, contributed to higher process yield than using traditional methods. The yield of the process is related to the water content of the samples being evaluated. It was observed that, as the temperature of the thermal process increased, the yield and water content in the salmon decreased (SV_57_ < SV_63_ < SP_100_ < RP_180_). The roasting due to the high temperature of the process (180 °C) was associated with higher weight losses related to water loss than during steaming (100 °C).

The obtained data is consistent with the research results of Brookmire et al. [26]. The water content in the roasted salmon evaluated by them decreased along with increasing temperature process (62.7–65.1%). Similar dependencies were also reported by other authors [17,36]. Conversely, in the studies by García-Linares et al. [19], the water content in sous-vide salmon (63.1%) was lower than in the cooked in the own sauce, which may result from high parameters of the sous-vide process (90 °C, 10 min). However, the opposite was achieved by these authors after cooking of rainbow trout with the same parameters.

Vaudagna et al. [37] suggested that the sous-vide method can be used to reduce water loss from meat. According to these authors, the main factor determining the level of culinary losses is the combination of time and temperature, while vacuum-sealed package plays a secondary role. This is also confirmed by the results of model tests carried out on salmon [20,36]. According to these authors, culinary losses increase with increasing temperature and heating time, and the highest weight losses are generated during the first 20–30 min of the process. This explains why the extended process time does not result in reduced yield.

### 4.2. Sensory Quality

Just a few studies comparing the sensory quality of sous-vide salmon with the processed and traditional methods have been performed. Research focused mainly on the sensory quality changes during chilled storage. Recently, a work devoted to seafood has been published. Sous-vide cooked mussels, in comparison with conventionally cooked mussels, were characterized by the highest overall scores, mainly because of its odor, flavor, and juiciness [25]. In turn, Zhang et al. [21] showed that the intensity of the carp soup flavor was the highest when cooked at 75–85 °C, while lowering process temperature to 55–65 °C resulted in its reduction. Too low parameters of the sous-vide process in abalone production was also associated with lower sensory scores, especially for flavor [27].

In our study, the low parameters of the sous-vide method (57 °C), despite a positive effect on higher juiciness of sous-vide in comparison with traditional methods, resulted in statistically significant (*p* ≤ 0.05) lower intensity of cooked fish odor and flavor, as well as a higher intensity (*p* > 0.05) of raw fish than salmon sous-vide prepared at 63 °C. Similar dependencies for tissue of slaughtered animals were obtained. As the process parameters increased, the hardness and the intensity of the cooked meat flavor increased, while the juiciness and off-flavor perception decreased [38,39,40,41,42].

The sous-vide salmon processed with pasteurization thermal dose obtained the highest notes of degree of liking. However, it did not differ significantly from the roasted in en papillote and steamed. Sous-vide salmon, wherein low process parameters were used (SV_57_) was significantly characterized by the lowest overall liking scores. This sample had the least-intense cooked fish flavor and odor, the least changed color and texture from fresh fish, which reflected in the lowest hedonic ratings of all mentioned attributes. Larsen et al. [22] suggested that salmon subjected to the least and the most intense parameters of thermal processing (poaching 100 °C, 3 min and deep frying) was the least liked. Heat treatment methods that induced in salmon a moderate intensity of color, smell and texture (oven baked salmon) were well accepted. Similarly, in the current study, sous-vide salmon prepared at 63 °C (SV_63_) and roasted “en papillote” had odor and flavor notes, as well as texture attribute intensities in middle range, hence, were liked the most. However, these were not statistically significant differences.

Larsen et al. [22] indicated that the taste and texture of the fish are key sensory characteristics affecting the general liking, while the overall appearance and odor have less impact. Similar findings were revealed in our study. Preferences for the texture of fish appear as early as preschool age. A study by Donadini et al. [43] revealed that the overall degree of liking is negatively affected by too much softness, jelly-like texture, fast melting, and easily falling apart texture, similar to that obtained in sous-vide (SV_57_) or steamed (SP_100_) salmon samples.

The color of salmon flesh is extremely important as the determinant of degree of doneness, nevertheless, lighter thermal treatment like in the SV_57_ case caused less appearance-liking. A higher L* value indicates a lighter color, which is desirable in order to ensure that the meat products will have high consumer acceptance [44]. There was a lack of significant (*p* > 0.05) differences in the overall liking between the roasted, steamed, and sous-vide method salmon prepared using parameters 63 °C for 80 min. This indicates different consumer preferences for the prepared salmon.

### 4.3. Color and Texture Instrumentally Evaluated

The color change of salmon during thermal treatment occurs in two stages: whitening and browning. The first stage occurs as a result of quick denaturation of heme proteins and carotenoid oxidation, while the second occur as a result of the Maillard reaction and protein-lipid reaction [17]. The browning phase was not observed in our own research, as in Ovissipour et al. [18], which may result from too low process parameters and the use of aluminum foil in the roasting process.

In this study, there is a tendency that the brightness of the salmon color increases with the increase in time and temperature of process, while decreases in redness and yellowness were observed. Similar dependencies were indicated by other authors [17,18].

The reverse trend in the SV_57_ case was observed, similar to the study of Picouet et al. [2]. In our own research and the mentioned authors’ research, the total color difference in relation to the raw material did not differ. In both cases, increase in values of a* and b* was stated. In the research of Ovissipour et al. [18], salmon was processed with sous-vide at 55 °C for 20 min, i.e., conditions similar to this experiment had similar values of color parameters in terms of L*, but lower values of a* and b*. However, difference between results may be caused by different raw material (diet, origin, species), so direct data comparison is not possible. Contradictory results were obtained by Larsen et al. [22], where the King Salmon processed with six various thermal treatments had lighter, redder, and more yellow color than the fresh one.

Correlations between sensory analysis results and instrumental texture parameters were reported [22]. In the case of this study, one could expect a correlation between the Warner-Bratzler test and the fragmentation ability. These assumptions were not confirmed by statistical analysis. The reason for this may be the complexity of the salmon tissue structure, as well as the temperature distribution in the material during thermal treatment. Surface layers of the sample are subjected to a much higher thermal dose than the internal ones. At the same time, several temperature- and time-dependent chemical and enzymatic changes occur, which affect the texture multi-directionally, thereby causing a large differentiation of material. The relatively highest correlation was found between the leakage intensity and the shear work of individual myosepts (r = −0.49, *p* ≤ 0.05). The leak accompanies protein coagulation and shrinkage, which results in increased shear work.

The reasons for salmon texture variation determined in instrumental texture assessment can be explained by protein changes. Proteins content correspond to the protein degradation cause by chemical and enzymatic activity. Although heating at 70 °C inactivates part of the muscle proteases, residual protease activity continues in the stored product. Heating up to 90 °C is necessary to prevent the texture of fish gels from deteriorating [45]. Cooking temperatures over 65 °C cause denaturation of myofibrillar proteins, which promotes meat toughening [46]. Kong et al. [17] observed the same pattern for heated pink salmon at 100–131.1 °C and explained the four sections as follows: rapid toughening, rapid tenderization, slow toughening, and slow tenderization. The denaturation of myosin in tuna begins at 35 °C, while the initial temperature of actin denaturation is about 58 °C and is completely denatured at 76 °C. Actin denaturation had a greater effect on texture changes, while myosin denaturation influenced the color and overall appearance of the product [4]. At a temperature of 50–60 °C (the largest increase in weight loss), the length of sarcomeres decreases, and the collagen begins to denature. At 60 °C, the space between the individual fibers is closed, and the myofibril shrinking begins. This may result from excretion of water from the protein-rich extracellular matrix [47]. Further heating leads to fibers shrinkage and a reduction in the water-holding capacity of the meat. The myofibrillar proteins are mainly responsible for this ability [48,49].

Table 7 summarizes the conclusions regarding the effect of various heat treatment method on the quality of salmon. The juxtaposition of the mean results show that the quality of sous-vide fish depends on the applied parameters. Too low parameters of the sous-vide process have a negative impact on their quality, while higher parameters result in high quality product, being the highest among those assessed.

## 5. Conclusions

Processing of salmon with the sous-vide method is more beneficial than roasting en papillote and steaming in pot in terms of given technological parameters, as evidenced by higher yield and water content in sous-vide products.

The sensory quality of sous-vide salmon depends on the time and temperature combination used. Salmon processed with sous-vide method—57 °C, 20 min (SV_57_ sample) had different sensory profile than other evaluated methods (sous-vide 63 °C, 80 min; steaming; roasting). Moreover, hedonic scores were lower than salmon prepared traditionally (*p* ≤ 0.05), as a result of low flavor intensity and texture attributes that differentiates them from the traditionally cooked fish (*p* ≤ 0.05).

In contrast, salmon made using higher parameters 63 °C for 80 min of sous-vide method (SV_63_), when compared with the traditional methods in terms of sensory quality had higher juiciness. Time and temperature combination is the main factor that determines the sensory quality of salmon. Moreover, using higher process parameters in the sous-vide method, it is possible to achieve a similar intensity of cooked fish odor and flavor without significant deterioration in the texture. The sensory quality of steamed salmon, roasted en papillote, and prepared using the sous-vide method (63 °C) was high and did not differ significantly. The lack of differences in the overall quality between the methods indicates that they are characterized by inherent sensory attributes.

These studies are important for the practical use of the sous-vide method in food service, wherein cooks often use very low temperature parameters. We suggest compensating the low process parameters with additional heat treatment (grilling, torching), which will add an attractive sensory value to the dish. It should be noted that fish prepared using the sous-vide method, processed, and served are at a lower temperature than those prepared using the traditional methods, so that the perceptibility of their aroma and flavor is lower.

## Figures and Tables

**Table 1:** Characteristics of evaluated salmon’ cooking methods.

Method	Process Conditions	Parameters of Process
Sous-vide	thermostable polyethylene-polyamide pouches (Hendi, Gądki, Poland). chamber type vacuum packaging machine (No 691310, Stalgast, Warsaw, Poland) sous-vide water bath (No 225448, Hendi, Gądki, Poland.) 1 fillet, 10 L of water.	Packaging: vacuum: 400 hPa vacuum time 60 s, sealing time 9 s. Cooking: SV_57_: at 57 °C for 20 min; SV_63_: at 63 °C for 80 min
Steaming (SP_100_)	steamer pot (φ24 cm) induction cooker (No: 770351, Stalgast, Warsaw Poland). 1 fillet, 1 L of water	Steam temperature—100 °C Power of induction cooker—400 W Process was performed until 70 °C* in the fillet core was reached (16 ± 2.5 min).
Roasting en papillote (RP_180_)	convection-steam oven (RedFox KE-423, RM Gastro, Veselí nad Lužnicí, Czech Republic) 1 fillet	Process was performed at 180 °C until a core 70 °C* was reached (23 ± 4.25min).

* parameters based on Health Canada [28].

**Table 2:** Water content and yield of process in salmon after heat treatment by different methods.

Parameter	Raw Material	$\mathrm{Heat}\;\mathrm{Treatment}\;\mathrm{Methods}\;\bar{x}$ ± SE (%)			
		Sous-Vide Method		Roasting en Papillote (RP_180_, 23 min)	Steaming SP_100_, 16 min)
		57 °C × 20 min (SV_57_)	63 °C × 80 min (SV_63_)		
Water content	62.9 ^a^ ± 0.6	62.8 ^a^ ± 0.6	61.2 ^a,b^ ± 0.7	58.9 ^c^ ± 0.7	60.7 ^b,c^ ± 0.7
Yield of process	-	93.7 ^c^ ± 0.6	90.9 ^b,c^ ± 0.5	83.8 ^a^ ± 1.6	88.4 ^b^ ± 1.1

SE—standard error; a, b, c—mean values marked by different letters in verses, differ significantly at *p* ≥ 0.05.

**Table 3:** Sensory profile of a salmon fillet processed with various heat treatment methods.

Attribute	Heat Treatment Method (*n* = 30)			
	Sous-Vide Method		Roasting en Papillote (RP_180_, 23 min)	Steaming (SP_100_, 16 min)
	57 °C, 20 min (SV_57_)	63 °C, 80 min (SV_63_)		
	Intensity (0–10 c.u.) $\bar{x}$ ± SE			
cooked fish odor	3.6 ^a^ ± 0.4	5.8 ^b^ ± 0.3	6.8 ^c^ ± 0.3	7 ^c^ ± 0.3
raw fish/seaside odor	2.6 ^a^ ± 0.4	2.8 ^a^ ± 0.3	2.7 ^a^ ± 0.3	3.5 ^a^ ± 0.5
fish oil odor	2 ^a^ ± 0.3	2.9 ^a^ ± 0.4	2.9 ^a^ ± 0.3	3.1 ^a^ ± 0.4
roasty odor	1.7 ^b^ ± 0.3	2.1 ^b^ ± 0.4	3.1 ^a^ ± 0.3	2.5 ^a,b^ ± 0.2
muddy/ musty odor	1.2 ^a^ ± 0.2	1.5 ^a^ ± 0.3	1.3 ^a^ ± 0.2	1.1 ^a^ ± 0.2
‘other’ odor	0.3 ^a^ ± 0.1	0.3 ^a^ ± 0.1	0.4 ^a^ ± 0.1	0.6 ^a^ ± 0.3
salmon color intensity	4.8 ^a^ ± 0.4	3 ^b^ ± 0.2	3.6 ^b^ ± 0.4	2 ^c^ ± 0.2
intensity of cellular juice and fat leakage	5.8 ^a^ ± 0.4	2.7 ^b^ ± 0.4	1.4 ^c^ ± 0.2	1.9 ^b,c^ ± 0.3
fragmentation ability (to myoseptum)	3.9 ^a^ ± 0.4	6.7 ^b^ ± 0.3	6.1 ^b^ ± 0.4	7.1 ^b^ ± 0.4
hardness	1.6 ^a^ ± 0.2	2.7 ^b^ ± 0.3	3.6 ^b,c^ ± 04	4.2 ^c^ ± 0.4
juiciness	7.6 ^a^ ± 0.3	6.7 ^a^ ± 0.3	5.2 ^b^ ± 0.4	5.6 ^b^ ± 0.3
cooked fish flavor	6.3 ^a^ ± 0.4	6.5 ^a,b^ ± 0.3	7.4 ^b^ ± 0.3	7.3 ^b^ ± 0.3
raw fish flavor	2.9 ^a^ ± 0.5	1 ^a^ ± 0.2	0.9 ^a^ ± 0.1	1.2 ^a^ ± 0.2
roasted fish flavor	1.9 ^a^ ± 0.3	3.4 ^b^ ± 0.4	3.8 ^b^ ± 0.4	3.6 ^b^ ± 0.4
salty taste	1.4 ^a^ ± 0.3	1.4 ^a^ ± 0.2	1.3 ^a^ ± 0.2	1.2 ^a^ ± 0.2
fatty flavor	3.7 ^a^ ± 0.4	3.5 ^a^ ± 0.5	3.4 ^a^ ± 0.4	2.6 ^a^ ± 0.4
‘other’ flavor	0.7 ^a^ ± 0.2	0.5 ^a^ ± 0.1	0.6 ^a^ ± 0.2	0.4 ^a^ ± 0.1
overall quality	6.9 ^a^ ± 0.2	7.3 ^a,b^ ± 0.2	7.3 ^a,b^ ± 0.2	7.8 ^b^ ± 0.2

SE—standard error; c. u.—conventional unit; a, b, c—mean values marked by different letters in verses, differ significantly at *p* ≤ 0.05.

**Table 4:** Hedonic scores of salmon fillet processed with various heat treatment methods in the opinion of consumers.

Heat Treatment Method		Odor	Appearance	Texture	Flavor	Overall Liking	Sum of Rank *
		Hedonic Scores (1–9 c.u.) $\bar{x}$ ± SE; *n* = 120					
Sous-vide method	57 °C, 20 min (SV_57_)	5.6 ^a^ ± 0.2	6.4 ^a^ ± 0.2	6.2 ^a^ ± 0.2	6.2 ^a^ ± 0.2	6.1 ^a^ ± 0.2	357 **
	63 °C, 80 min (SV_63_)	6.6 ^b^ ± 0.2	7.2 ^b^ ± 0.1	7.2 ^b^ ± 0.1	7.3 ^c^ ± 0.1	7. 3^b^ ± 0.1	270
Roasting en papillote (RP_180_, 23 min)		6.7 ^b^ ± 0.2	6.9 ^a,b^ ± 0.1	7.1 ^b^ ± 0.1	7.1 ^b,c^ ± 0.2	7.1 ^b^ ± 0.1	258
Steaming (SP_100_, 16 min)		6.7 ^b^ ± 0.1	6.7 ^a^ ± 0.1	6.9 ^b^ ± 0.1	6.8 ^b^ ± 0.1	6.8 ^b^ ± 0.1	315

c.u.—conventional units; SE—standard error; a, b, c—mean values marked by different letters in verses, differ significantly at *p* ≤ 0.05; *—sum of sensory ranks obtained by ranking method, ** *p* = 0.001.

**Table 5:** Color comparison of salmon of raw and processed with various heat treatment methods.

Color (L* a* b*)	Raw	Heat Treatment Method ($\bar{x}$) ± SE			
		Sous-Vide		Roasting (RP_180_, 23 min)	Steaming (SP_100_, 16 min)
		57 °C, 20 min (SV_57_)	63 °C, 80 min (SV_63_)		
Lightness L*	45.41^a^ ± 1.13	68.07 ^b^ ± 0.59	78.85 ^c^ ± 0.48	79.34 ^c^ ± 0.94	79.09 ^c^ ± 0.64
ΔL	-	23.9 ^a^ ± 0.4	34.09 ^b^ ± 1.11	32.42 ^b^ ± 1.05	33.17 ^b^ ± 0.54
a*	20.82 ^a^ ± 0.8	22.04 ^a^ ± 0.59	16.4 ^b^ ± 0.52	13.81 ^b^ ± 1.34	15.25 ^b^ ± 0.98
Δa	-	1.71 ^a^ ± 0.32	−4.32 ^b^ ± 1.02	−7.17 ^b^ ± 0.85	−5.59 ^b^ ± 0.5
b*	22.7 ^a^ ± 1.16	28.34 ^b^ ± 0.94	20.81 ^a^ ± 0.6	19.42 ^a^ ± 1.15	21.02 ^a^ ± 1.25
Δb	-	6.14 ^b^ ± 0.48	−1.5 ^a^ ± 1.88	−4.14 ^a^ ± 1.92	−1.83 ^a^ ± 1.98
ΔC	-	6.38	4.57	8.28	5.88
Chroma C*	30.8	35.9	26.79	23.85	25.97
Total color difference (ΔE)	-	24.73 ^b^ ± 2.34	34.4 ^a^ ± 1.08	33.47 ^a^ ± 1.53	33.69 ^a^± 0.95

SE—standard error; a, b, c—mean values marked by different letters in verses, differ significantly at *p* ≤ 0.05.

**Table 6:** Texture of salmon processed with various heat treatment methods.

Parameter	Type of Test	Heat Treatment Method ($\bar{x}$) ± SE			
		Sous-Vide		Roasting (RP_180_, 23 min)	Steaming (SP_100_, 16 min)
		57 °C, 20 min (SV_57_)	63 °C, 80 min (SV_63_)		
Shear force (N)	of fish cube (W-B)	5.9 ^a,b^ ± 0.5	3.9 ^a^ ± 0.3	5.8 ^a,b^ ± 0.6	6.9 ^b^ ± 0.6
	of single myoseptum (C-K)	8.1 ^a^ ± 0.8	12.2 ^b^ ± 0.5	10.8 ^b^ ± 0.6	8.1 ^a^ ± 0.5
Shear work (N·mm)	of fish cube (W-B)	63.8 ^a^ ± 5.4	56.8 ^b^ ± 2.3	69.5 ^c^ ± 5.4	75 ^d^ ± 3.9
	of single myoseptum (C-K)	12.1 ^a^ ± 1.3	36.3 ^b^ ± 1.2	28.7 ^c^ ± 1.2	18.5 ^d^ ± 1

W-B—Warner-Bratzler attachement; C-K—Craft Knife; SE—standard error; a, b, c, d—mean values marked by different letters in verses, differ significantly at *p* ≤ 0.05.

**Table 7:** Salmon quality changes depending on the cooking method.

Quality Attributes	Heat Treatment Method			
	Sous-Vide Method		Roasting (RP_180_, 23 min)	Steaming (SP_100_, 16 min)
	57 °C 20 min (SV_57_)	63 °C 80 min (SV_63_)		
**Technological Quality**				
yield	++	+	–	+
water content	-	+	++	+
**Sensory Quality**				
intensity of odor **odor -liking	– –	+ +	++ ++	++ ++
appearance-liking	–	++	+	+
intensity of flavor**	–	+	++	++
flavor-liking	–	++	+	+
hardness* juiciness texture-liking	++ ++ –	+ + ++	+ + +	– – +
overall quality	–	+	+	++
overall liking	–	++	+	+
color changes ΔE	++	0	0	0
force and work shear	+	++	+	–
**Sum**	**0**	**+18**	**+15**	**+11**

** typical for cooked fish; ++, the mean value of given thermal treatment is the highest; +, the mean for given sample has intermediate values; –, mean of given sample has the lowest values; 0, no tendency.
